# Liquefied Atmospheric Air

**Published:** 1898-04

**Authors:** 


### LIQUEFIED ATMOSPHERIC AIR.

Now and again, of late years, the scientific journals have referred to liquid air,—that is, atmospheric air so condensed as to assume the liquid form. The possibility of this, as a laboratory experiment, has long been demonstrated; but the conditions required to bring it about were so exacting that, until very recently, it has been a curio of the physical laboratory. Heretofore an extremely low temperature, difficult and costly to secure, has been employed in producing liquefied air; the cost is said to have been about twenty-five hundred dollars a quart, and the quantity obtained so small that its peculiar properties could be but imperfectly examined.

Very recently, Mr. Charles E. Tripler, of New York, has solved the problem of producing cheaply and on a commercial scale liquefied atmospheric air by an ingenious and simple process. The product is as yet too new to permit an estimate of its possible usefulness.

Mr. Tripler first, by ordinary means, condenses the air to a pressure of two thousand pounds to the square inch. This he permits to flow through and around a copper worm, and through an opening the size of a fine needle. It expands as it issues from this orifice, and cools very considerably. This cooled air is passed through another coil, which cools it to a still lower temperature, and, escaping, it passes through a third coil, which cools it down to below minus 191° Centigrade, or 320° below zero by the Fahrenheit scale. It runs out of this third coil in a liquid stream as large as the finger, without any pressure at all. This extremely low temperature is produced entirely by the expansion of the air itself.

On Thursday, January 27, 1898, Dr. Barker received at his laboratory at the University of Pennsylvania, from New York, a "milk-can" full of this liquefied air, and before an interested audience demonstrated some of its physical properties. It was the first time, indeed, that any opportunity had been offered to exhibit this interesting product so freely. Heretofore liquefied air has been confined in strong steel cylinders, under heavy pressure, or in glass tubes kept at an extremely low temperature, and in very small quantities. In this case, two and a half gallons, which were used in the demonstration, were brought from New York in a common milk-can, packed with felt, and with a protecting air-space, but were not under pressure.

Dr. Barker, in explaining the principles which lie at the base of this discovery, said, "Matter exists in three states,—solid, liquid, and gaseous,—dependent upon its temperature, pressure, and consequent volume. Compress a gas and it becomes denser; but so long as it remains a gas it is not converted into a liquid. If we condense a vapor it becomes a liquid by pressure at a given temperature. The doctor illustrated this by reference to water in a receiver exhausted of air. The water evaporates and fills the receiver with vapor, and there is then liquid and vapor there in the presence of each other. If heated, the amount of vapor will increase. The boiling-point of water is 100° C. If the water is heated to that point, the pressure of vapor in that receiver is just equal to the barometric pressure. If the temperature is raised above that point, the water boils and becomes vapor; at that point it remains liquid. Applying that principle to the air, which is already in a gaseous state, imagine a point where it is on the balance, as in the above example, where a lowering of temperature makes it a liquid and a rise of temperature makes it gaseous. That point is a temperature of minus 191° C., or, by the Fahrenheit scale, 320° below zero.

"At this point air liquefies and remains a liquid at barometric pressure the same as water. If raised above that temperature, as by standing open to the atmosphere, it evaporates, the evaporation tending to keep it cool, so that a tumblerful of the liquid would all evaporate in about half an hour. Nitrogen liquefies at minus 193° C. and oxygen at minus 180° C. It is evident, therefore, that, as the liquid becomes warmer, the nitrogen will pass off into the gaseous state, leaving a greater portion of oxygen behind to evaporate at a still higher temperature. It thus follows that, in time, the liquid becomes principally oxygen; this is shown by the bluish color which it gradually assumes after standing open awhile."

The doctor then proceeded to experiment with this new form of air, showing some of its peculiar properties. When first poured into a tumbler, the liquid would boil until the glass had cooled down. This was because any rise of temperature above minus 191° C. would, as before described, raise the liquid above its boiling-point. Any substance whatever dipped into the liquid had the same effect, and made it boil. Its vapor coming in contact with the air cooled the moisture in the air, which fell down in a cloud of hoar frost. A piece of tin, also a piece of flexible rubber tube, thrust into the liquid, became quite brittle. Neither copper nor platinum were at all affected. Alcohol was frozen into a solid chunk by pouring a quantity into the liquid; mercury was frozen solid by pouring some of the liquid upon it, and was then used to drive a nail into a piece of hard wood. A taper, extinguished, but having a spark on its end, broke into a brilliant flame when placed over the liquid, showing the presence of the evaporating oxygen at its surface. Many other experiments were performed, which kept the audience in a state of breathless wonder, not only at what they saw, but at the possibilities which may soon become realities in the economic use of this new form of air.

